# Human Monogenic Disease Genes Have Frequently Functionally Redundant Paralogs

**DOI:** 10.1371/journal.pcbi.1003073

**Published:** 2013-05-16

**Authors:** Wei-Hua Chen, Xing-Ming Zhao, Vera van Noort, Peer Bork

**Affiliations:** 1 European Molecular Biology Laboratory (EMBL) Heidelberg, Heidelberg, Germany; 2 Department of Computer Science, School of Electronics and Information Engineering, Tongji University, Shanghai, China; 3 Max-Delbrück-Centrum für Molekulare Medizin (MDC), Berlin-Buch, Berlin, Germany; Max-Planck-Institut für Informatik, Germany

## Abstract

Mendelian disorders are often caused by mutations in genes that are not lethal but induce functional distortions leading to diseases. Here we study the extent of gene duplicates that might compensate genes causing monogenic diseases. We provide evidence for pervasive functional redundancy of human monogenic disease genes (MDs) by duplicates by manifesting 1) genes involved in human genetic disorders are enriched in duplicates and 2) duplicated disease genes tend to have higher functional similarities with their closest paralogs in contrast to duplicated non-disease genes of similar age. We propose that functional compensation by duplication of genes masks the phenotypic effects of deleterious mutations and reduces the probability of purging the defective genes from the human population; this functional compensation could be further enhanced by higher purification selection between disease genes and their duplicates as well as their orthologous counterpart compared to non-disease genes. However, due to the intrinsic expression stochasticity among individuals, the deleterious mutations could still be present as genetic diseases in some subpopulations where the duplicate copies are expressed at low abundances. Consequently the defective genes are linked to genetic disorders while they continue propagating within the population. Our results provide insight into the molecular basis underlying the spreading of duplicated disease genes.

## Author Summary

Duplicated genes, as opposed to singletons, are genes that have additional copies in a genome due to evolutionary mechanisms such as whole genome duplication, homologous recombination or retrotransposition events. Duplicates can have similar functions and thus mask the phenotypic consequences when one copy is mutated. Conversely, the corresponding phenotypes would manifest themselves when mutations occur in singletons, since functional compensation is rare among non-duplicated genes. It would thus be expected that the primary source of monogenic diseases, diseases caused by mutations within a single gene, is singletons. However, the opposite was found to be true. Additionally, we found the functional similarity of duplicated disease genes to be greater than that of duplicated non-disease genes of an equivalent duplication age. So how could the stronger functional compensation among duplicates increase their likelihood to associate with diseases? We propose that due to functional compensation in duplicates, disease-causing mutations are less likely to be removed from a human population in large scale since the phenotypes are masked; however, the functional compensation could be lost in a subpopulation, perhaps due to intrinsic variation in gene expression, and therefore lead to diseases. As a result, the duplicated disease genes are linked to genetic diseases, yet they continue to spread within the human population.

## Introduction

Elucidating the molecular basis of human genetic disorders is one of the most important tasks in medical biology. The availability of the human genome sequence [1], [2] has facilitated the identification of individual disease genes, e.g. in family pedigree analyses [3] as well as genome-wide association studies (GWAS) [4], [5]. Exploring the characteristics of known disease genes and differences from non-disease genes using bioinformatics methods in recent studies has provided, for example, knowledge of their function [6], evolutionary origin [7], [8], selective constraints [9]–[11] and network properties in the protein-protein interaction (PPI) network [12]–[14], and insights into the genetics underpinning human inherited disorders, facilitating *in silico* identification of novel disease genes [9], [11].

However, recent studies have revealed some controversial findings related to duplicated genes and no clear explanation has been given so far. For example, the accepted hypothesis was that disease genes tend to be singletons with fewer paralogs [15] since duplication can lead to functional redundancy [16]–[18] and thereby mask the effect of deleterious mutations [15], [19]; however, disease genes were found surprisingly enriched in duplicates [8]. Moreover, the molecular mechanism by which the duplication statuses of disease genes contribute to their increased presence in the human genome is still unclear. Recently, it has been proposed that the presence of duplicates permits the accumulation of disease-causing mutations, the emergence of disease genes thus would be more likely to associate with duplicates [8]. Here we argue that this line of reasoning does not necessarily predict the enrichment of disease genes in duplicates even when the compensational capacity between duplicates is considered. For example, in duplicates (i.e. more recent ones) whose functional redundancy is resilient enough to mask some disease-causing mutations in one of the copies, the proportion of disease genes would be lower compared with that of overall singletons; however, for duplicates (i.e. older ones) whose compensation capacity is partial or no longer effective, they would be purged from the human genome at the same rate as singletons; combined together, the overall proportion of disease genes in duplicates would still be lower. Summarizing recent literature, we realized that the duplication-functional redundancy theory alone is perhaps insufficient in explaining the observed enrichment of disease genes in duplicates, and the contribution of additional factors should be explored and taken into consideration.

In this work, we sought to provide a clear illustration on the evolutionary forces governing the propagation of disease genes in the human population by surveying exhaustively the characteristics of disease genes and comparing those with non-disease genes. We focused on monogenic disease genes (MDs) that have a clear association with and contribution to human genetic disorders, and tried to address the following questions. First, can the enrichment of disease genes in duplicates first revealed by Dickerson et al [8] be reproduced in an updated dataset and what are the contributions of multiple paralogs in multi-gene families? Second, if disease genes indeed tend to have functional backups, is this supported by evidence showing a higher functional similarity between paralogs of disease genes than paralogs of non-disease genes? A key factor being, if the functional divergence of disease genes is greater than that of non-disease genes, a lower or comparable proportion of disease genes in duplicates would be expected, mimicking a behavior that of singletons. Due to the divergence of the functional redundancy of duplicated genes [20] stratification of the genes according to their duplication age was necessary, otherwise resulting in false conclusions as shown in [21]. Third, what are the evolutionary factors acting on human disease genes within and/or across species that could contribute further to the functional compensation of duplicated disease genes? And finally, what are the molecular mechanisms underlying the spreading of disease genes as duplicates or singletons in the human population? In other words, how could the functional redundancy between duplicates actually increase their likelihood of being disease genes?

## Results

### Disease genes are enriched in duplicated genes

**Figure 1:**
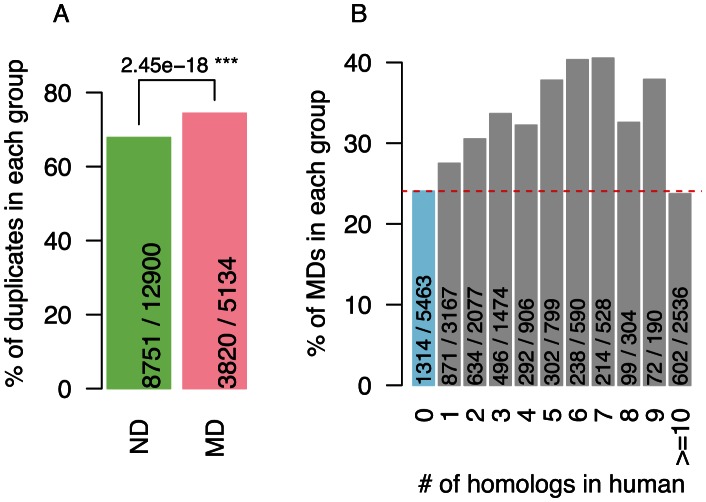
Duplicated genes are enriched in monogenic disease genes. A) percentages of duplicates in monogenic disease genes (MD) and non-disease genes (ND). B) percentages of monogenic disease genes as function of number of duplicates in human; 0 indicates that genes are singletons. Here duplicates were defined using TreeFam. P-value shown in panel A was calculated using Fisher's Exact Test; level of significance: *** <0.001, ** <0.01, * <0.05. Numbers shown within the bars are gene counts (subset/total).

Initially, we investigated the duplications of human disease genes. Here, we considered three widely used approaches to detect duplicated genes in the human genome, including those based on simple homology (FASTA), gene family evolution (TreeFam) and orthology (eggNOG v3) (see Methods) which resulted in similar conclusions for all methods (Figures S1 and S2). As shown in Figure 1, we found that 55% monogenic disease genes (MDs) were duplicates, a significantly higher fraction than in non-disease genes (NDs; *p* = 2×10^−8^; Fisher's Exact Test); similarly, we found 23% of the duplicates are also MDs, compared to 18% in singletons (Figure 1B; see also Dataset S1). Since duplicates are often found to be functionally compensating [16], our results suggest disease genes are enriched in functional backups. Strikingly, we found that the number of paralogs in the same gene family did not have a significant impact on gene disease status (Figures 1B, S1B and S2B), suggesting non-additive functional compensation from multiple gene family members.

### Functional redundancy in duplicated disease genes

We next sought to find additional evidence for functional redundancy in duplicated disease genes by comparing with duplicated non-disease genes. Since the functional redundancy between duplicates decreases over time [20], it is essential to compare duplicates of a similar age. We therefore first divided duplication pairs (gene-closest paralog) into distinct groups according to their duplication age, and then divided them into disease gene containing pairs, if at least one gene in a pair is disease-related (MD-pairs), and non-disease gene containing pairs otherwise (ND-pairs) (see Methods).

#### Evidence from unbiased datasets

We first of all analyzed the differential expression patterns and sequence divergences between duplicated genes, which are widely believed to be important indicators of functional similarities [22].

**Figure 2:**
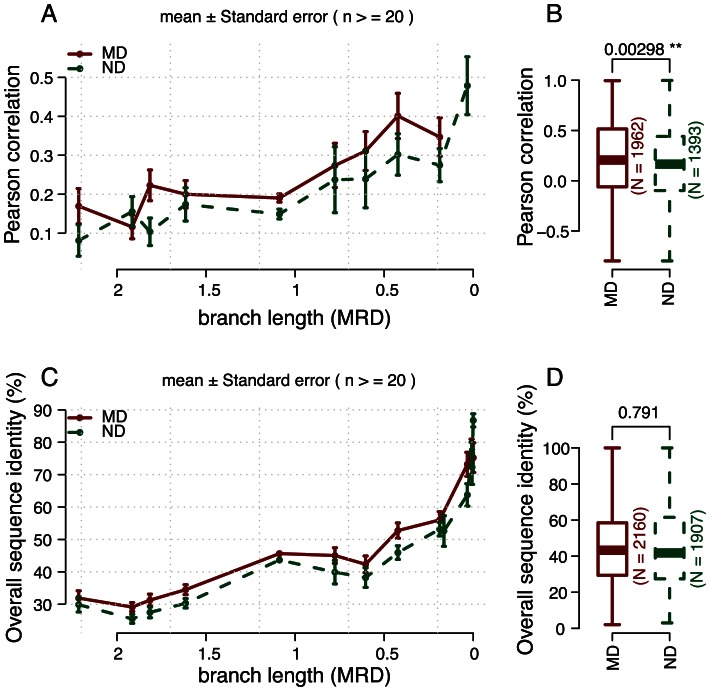
Evidence for functional redundancy in duplicated disease genes. Comparing with duplicated non-disease genes (ND) of similar duplication age (represented by branch length, see Methods), monogenic disease genes (MD) tend have A) higher co-expression co-efficient (p-value = 1.69×10^−3^, Hypergeometric Distribution test), C) higher sequence similarity (p-value = 1.66×10^−3^, Hypergeometric Distribution test). Results in A) can be repeated using another set of gene expression data (Figure S3). P-values shown in the boxplots (B and D) were calculated using two-sample Wilcoxon Rank Sum Test; see Materials and Methods for more details regarding the statistical tests. Numbers shown next the boxplots are the numbers of valid samples (after removing samples with missing values).

Using gene expression profiles in 36 human normal tissues obtained from [23], we found that the co-expressions between MDs and their closest paralogs are in general higher than that of non-disease genes of similar duplication age (Figure 2A; duplication age delineated by the total branch length from the node representing where the duplication event happened on the species tree to the leaf node of human; see Methods); this is also true when ages are omitted (Figure 2B). Additionally, we found the co-expressions tend to decrease with increasing duplication age, consistent with previous studies [20]. The same results can be obtained using the expression data from [24] (Figure S3).

Similarly, we found that the protein sequence identities of MD-pairs are higher than that of ND-pairs of similar age. Similar to the co-expression results, the sequence identity in general correlates negatively with the divergent time, as shown in Figure 2C. Thus in both datasets we obtained consistent results indicating higher functional similarities between monogenic disease genes and their closest paralogs than for ND paralog pairs. Since all genes in the two datasets are either present (e.g. protein sequences) or have an equal possibility to be present (e.g. gene expression data from microarrays), we considered the two datasets unbiased.

#### Additional evidence from biased/incomplete datasets

We then compared the characteristics between MD- with ND-pairs using two additional datasets, namely Gene Ontology (GO) and human physical protein-protein interactions (PPIs). GO annotations are known to be biased towards highly expressed and more conserved genes [25]; the same would also apply to the PPI data. Additionally, current GO annotation and human PPI network only cover limited numbers of genes; consequently, only ∼37% duplication pairs were annotated by GO, and ∼36% by PPIs. We thus considered the two datasets biased.

**Figure 3:**
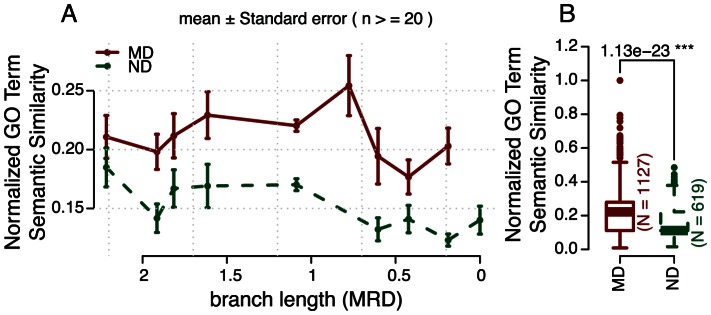
Evidence for pervasive functional redundancy in duplicated disease genes based on Gene Ontology annotations. Compared with duplicated non-disease genes (ND) of similar duplication age (represented by branch length, see Methods), monogenic disease genes (MD) tend to have A) higher functional similarity according to Gene Ontology annotations with their most recent duplications (MRDs; p-value = 7.77×10^−5^, Hypergeometric Distribution test); B) the same are also true when duplication ages are omitted (Wilcoxon Rank Sum Test).

We obtained GO annotations for human gene products from Ensembl Biomart and used the Bioconductor package GOSemSim [26] to measure semantic similarities between GO terms associated with duplicate genes (see Methods). In light of recent discussions on possible biases in GO and wrong interpretations of the results due to the biases [27], [28], we tested whether disease and non-disease genes were equally represented in the GO annotations. We found MD genes were significantly better annotated by GO and associated with more GO terms (*p* = 2.36×10^−32^, Wilcoxon Rank Sum Test; Figure S4); the GOSemSim value measured on a pair of duplicated genes is inversely correlated with the maximum number of GO terms of individual genes associated with in a pair (Pearson's correlation: *p* = 1.12×10^−35^, R = −0.27). We therefore adopted a normalized version of GOSemSim as an approximation for functional redundancy. As shown in Figure 3, we found disease genes tend to have similar functions with their closest paralogs compared with that of non-disease genes of similar age (Figure 3A); the same results could be obtained when age was omitted (Figure 3B).

Similarly, by calculating the percentage of shared PPI partners between duplicates, we found disease genes also tend to have higher functional similarity with their duplicates than non-disease genes (Figure S5). Thus all the datasets generated consistent results, thereby providing extensive evidence for the pervasive functional redundancy by duplicates for human monogenic disease genes.

### Higher purifying selections on duplicated disease genes

**Figure 4:**
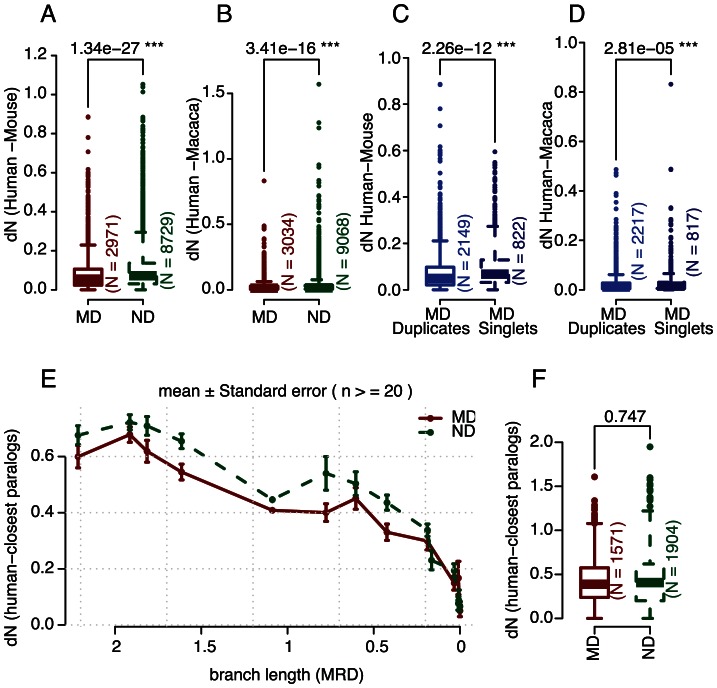
Higher purifying selections on duplicated disease genes. Compared with non-disease genes (NDs), disease genes tend to have lower dN values with their mouse- (A) and Macaca- (B) one-to-one orthologs. Furthermore, compared with disease singletons (singlet genes or singletons refer to those that do not share significant protein sequence similarities with other human genes), duplicated disease genes tend to have lower dN values with their mouse- (C) and Macaca- (D) orthologs. The higher selective constraints on duplicated disease genes can be also seen within the human genome; for example, compared with duplicated non-disease genes (ND) of similar duplication age, disease genes tend to have lower dN values with their closest paralogs within human (E; p-value = 4×10^−7^, Hypergeometric Distribution test). However the same isn't true when age is omitted (F), highlighting the importance of dividing gene pairs according to their duplication age. P-values shown in the boxplots (A∼D and F) were calculated using two-sample Wilcoxon Rank Sum Test. A similar plot showing no outliers is also available in Figure S6.

Previous studies suggested that disease genes were under purifying selections compared with non-disease genes, by measuring the numbers of nonsynonymous substitutions per nonsynonymous site (dN) between human-mouse orthologs [11]. We confirmed these observations in our dataset using one-to-one orthologs between human and mouse, as well as those between human and macaque; the results are shown in Figure 4A and 4B, respectively. Furthermore, we found the selective constraints on disease duplicates are higher than on disease singletons (genes that do not have homologs in the human genome), as shown in Figure 4C and 4D.

The higher purifying selection on duplicated disease genes can also be observed within the human genome; as shown in Figure 4E, we found that MD- pairs always have lower dN values than ND-pairs of similar age.

## Discussion

In summary, we have made two interesting observations regarding disease genes in duplicates. First, we have shown that human monogenic disease genes tend to frequently have functionally redundant paralogs, by comparing their characteristics to that of non-disease genes, stratifying both categories according to duplication age. Second, duplicates tend to harbor more disease genes than singletons, confirming the observation by an earlier study [8], but contradicting theoretical expectations.

What are possible explanations for these observations? A possible scenario is that a disease gene and its duplicate are simultaneously required for certain functions; for example, they might be involved in the same protein complex. In this case, the two genes would be highly co-expressed and evolve similarly. However this is unlikely because the so-called “balance hypothesis” – both underexpression and overexpression of protein complex subunits would lower fitness of the host organism – [29] predicts that 1) duplicates are rarely involved in protein complexes and 2) the two duplicates from a common ancestor are rarely retained by the same complex unless all other members of the complex are also duplicated and the extra copies are also retained; otherwise the protein complex is imbalanced and evolutionarily deleterious [29]. We found that the first held true in MDs as well as NDs in human using a protein complex dataset from [30], and comparing them with non-disease genes. Disease genes and their closest paralogs are significantly less likely to be involved in the same complexes (*p* = 0.0002, Odds Ratio = 0.57; Fisher's Exact Test). These results are consistent with a previous study in which only one gene out of a pair of duplicates was found to be associated with diseases [8]. A previous study suggested that duplicates associated with whole genome duplications (WGDs) are dosage balanced [31] and thus might not abide by the balance hypothesis. However, we found that pairs of WGD duplicates do not have a high likelihood to be in the same complexes compared with pairs of duplicates associated with small scale duplicates (SSDs) (*p* = 0.22, Fisher's Exact Test); similar results could be obtained (*p* = 0.63; Fisher's Exact Test) using protein complex data from a genome-wide experimental survey on soluble proteins in human [32]. Thus, WGD is not a confounding factor for our observation.

**Figure 5:**
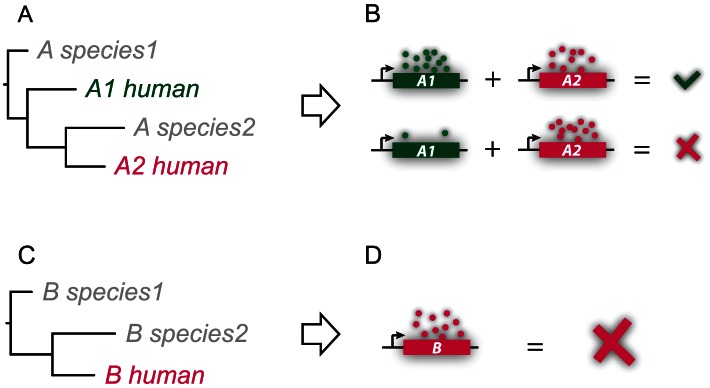
A model for the effect of functional compensation on the propagation of duplicated disease genes in the human population. This model is based on two previous experimental studies. The first showed that genes with identical promoters could have very different expression abundances in individual *E. coli* cells [33]. The second showed different *C. elegans* individuals carrying the defect gene could demonstrate varying phenotypes ranging from wild type to stalled development on embryogenesis, depending on the expression abundance of a duplicate gene [34]. We therefore propose that in cases where a duplicate (A1_human) exists (panel A), the functional impairment caused by mutations on a disease gene (A2_human) could be compensated; however due to intrinsic expression stochasticity of the duplicate copy, some individuals would appear to be normal while some others show reduced fitness (panel B). Consequently this gene A2 is linked to genetic disorders while the deleterious mutations it carries continue to spread instead of being removed in the human population. On the other hand, if a disease gene (B_human; panel C) is a singlet without any paralogs, its mutations then would be more likely to be purged from the population (panel D) since compensation by non-duplicates via genetic interactions is relatively rare [16], [17].

So how could functional redundancy actually promote the enrichment of disease genes in duplicates? Here we propose a new model. We argue that functional compensation by duplication of genes would help mask the phenotypic effects of deleterious mutations, as previously suggested, and reduce the probability of purging the defect genes from the human population. The functional compensation could be further enhanced by the higher purifying selection on duplicated disease genes within and between species. However, due to the intrinsic expression stochasticity among individuals [33], [34], the deleterious mutations could present as genetic diseases in subpopulations where the duplicate copies express in low abundances. In other words, the corresponding genes would manifest as disease genes, while the mutant allele would remain in the population instead of being removed. This model is illustrated in more details in Figure 5. Consequently, duplicates would be enriched in disease genes; the enrichment is weak, albeit significant, due to the complexity of gene regulation in the human genome.

## Materials and Methods

### Human genes and sequences

We obtained 21,731protein coding genes and the corresponding protein and coding sequences (CDS) from Ensembl [35] version 59. In cases one gene coding for multiple proteins, the longest protein and the corresponding CDS is chosen as representative.

All other gene annotations such as HGNC symbols, NCBI gene IDs and accession numbers were mapped to Ensembl gene identifiers to facilitate data integration. We downloaded the mapping data using Ensembl BioMart.

### Disease genes

We collected human disease genes from OMIM [36] and two recent literatures [37], [38]. In each of the sources disease genes were divided into two categories, MDs – those associated with monogenic diseases, and PDs – those associated with polygenic diseases. We assigned genes associated with both types of diseases into the MD group; please note that changing this definition, for example by assigning this type of genes into the PD group did not change our main conclusions (see Dataset S1).

All other genes that are not included in any of the three sources are considered non-disease genes (NDs).

### Duplicated genes

We used three approaches to find duplicated genes in the human genome, including methods based simple homology search (FASTA), gene family evolution (TreeFam [39]) and orthology (eggNOG3 [40] using euNOG).

Using the homology-based method, if two human genes had a bitscore higher than 80 in a FASTA [41] search at protein level, and the aligned region covers at least 50% of the shorter protein, they are considered as duplicates; please consult ref [21] for more information about the chosen cutoffs. Changing the cutoffs, for example by increasing the required proportion of the aligned regions for homology detection did not affect our results; see Figure S1 for more details.

In the latter two methods, if a gene family or an orthologous group contains two or more human genes, these genes are duplicates. The numbers of duplicated genes identified by the three methods are 14,014, 14,084 and 11,853, respectively.

### Dating duplication events on species tree

We downloaded all gene families as well as their corresponding phylogenetic trees from TreeFam [39] ver8.0. We excluded gene families that do not contain human genes, or contain genes from less than four different species, resulting in a set of 9,643 gene families.

For each pair of duplicates in a gene family, we dated the (putative) duplication event by comparing the topology of the corresponding gene tree with that of a species tree. To compare with the TreeFam gene trees, we used a species tree downloaded from Ensembl (http://www.ensembl.org/info/docs/compara; see also Figure S8).

As shown in Figure S7, to date a duplication event of a pair of duplicated genes (A2 and A3 in this case; see Figure S7A), we first located their last common ancestor (LCA) on the gene tree, and collected all the genes that are descendent to this LCA (Figure S7A; in this case A_rat, A_mouse, A2_human and A3_human) and their corresponding species (in this case human, mouse and rat); then we mapped these species on to the species tree (Figure S7B) and located the corresponding LCA; the age (divergent time) of the duplication event was then defined as the total branch length from this LCA to human on the species tree.

The trees shown in Figures S7 and S8 were visualized and prepared using online tools, iTol [42] and EvolView [43].

### Identifying duplicates associated with whole genome duplications (WGDs)

Two rounds of whole genome duplication (WGDs) occurred during early chordate evolution [44], [45]. Duplicated genes for which their duplication events can be dated back to that time are thus likely to associated with WGDs. Using similar criteria to [31], we were able to identify in total 6,560 genes with their most recent duplication (MRD) ages dated after the split of human and *Ciona intestinalis* (Ascidian), and before the split of human and fishes including *Takifugu rubripes* (see also Figure S8); we found this number of WGD associated duplicates remarkably similar to that of [31] although different methods and numbers of species were used.

### Gene expression profiles in normal tissues

We obtained the expression profiles of human genes in normal tissues from two sources [23], [24]; we were able to map 12,436 and 17,553 probe-sets to Ensembl 59 gene IDs for the two expression datasets, respectively. Both datasets generated similar results. Therefore we showed the results based on [23] in the main text; results based on [24] are shown in Figure S3.

### Gene ontology (GO) analyses

We downloaded GO annotations of human gene products from Ensembl BioMart and GO term hierarchy file ‘gene_ontology_ext.obo’ (format version 1.2; Feb 2012) from the Gene Ontology database [46]. Genes (gene products) without GO annotations were excluded from further analyses.

To compare functional redundancy based on semantic similarity of GO terms between any given two genes, we used the Bioconductor package GOSemSim [26]and restricted our analyses on leaf-GO terms in “molecular function”. Due to known biases towards a better annotation for disease genes (see Results), we adopted a normalized version of GOSemSim as the following formula:
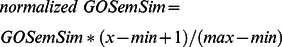
where ‘*x*’ is the maximal number of GO terms associated with individual genes in a duplication pair, ‘min’ is the minimal number of GO terms associated with genes, ‘max’ is the maximal number of GO terms associated with genes; ‘+1’ is used to avoid zeros.

### Protein-protein interaction data

We collected the protein-protein interaction data from several public databases, including STRING [47] (version 9, score> = 0.7), HPRD [48] (June 29, 2010), DIP [49] (Feb 28, 2012), MINT [50](Feb 6, 2012), IntAct [51] (Feb 7, 2012), and BioGRID [52] (version 3.1.82), and considered only physical bindings. In addition, we also included one experimental dataset [53] and one curated dataset from the literature [54]. In total, we obtained 80,202 interactions among 12,839 gene products.

### dN values

For each pair of duplicates in the human genome, we used a KaKs_Calculator [55] tool to calculate the dN (the numbers of nonsynonymous substitutions per nonsynonymous site).

We also downloaded dN values between human genes and their homologs in mouse and macaque from Ensembl [35] BioMart; we retained entries with “Homology Type” of “apparent_ortholog_one2one” or “ortholog_one2one”.

### Statistical tests

In this study we applied three statistical tests to different types of datasets. 1) Fisher's Exact Test. We used it to test whether monogenic disease genes (MDs) are more likely to be duplicates compared with non-disease genes (NDs). Since genes can be divided into four groups according to two kinds of classifications (association with diseases and being duplicates), it is suitable to use Fisher's test. 2) Wilcoxon Rank Sum Test. We used this test to compare two sets of numerical values (for example two sets of dN values for MD and ND genes respectively) and access whether one tends to have higher values than the other; in this study it was often associated with boxplots. 3) Hypergeometric Distribution Test. To test whether duplicated MD genes tend to have higher functional redundancy with their most recent duplicates than that of ND genes of similar age, each of the two groups would be further divided into more than 10 age groups. We found in all cases, the majority of the MD groups had higher (or lower) mean values than the ND groups of the same age (for example Figure 2A). To check whether such observations were significantly different from random expectation, we applied the Hypergeometric Distribution Test using the following function in R: phyper(q, m, n, k), where m refers the number of cases where the mean values of the MD groups are higher (or lower) in the pool, n refers the number of cases where the mean values of the MD groups are lower (or higher) in the pool, k refers the number of cases randomly chosen from the pool of m + n, and q refers to the number of cases out of k where the mean values of the MD groups are higher (or lower). In this study we set m = n = k = the number of valid age groups. All tests were performed using R (http://www.r-project.org/).

### Availability of the materials and methods

All raw data and R scripts used in this study are available in Dataset S1 as an archive file; also included in this archive is a detailed instruction for the readers to reproduce our main results, including all the figures, supplementary figures, and statistical tests except Figure 5, which was plotted manually.

## Supporting Information

Figure S1 Similar to Figure 1, only the duplicated genes were detected using FASTA. Here we also tested the impact of different cutoffs of aligned regions required for homology detection on our results; four cutoffs were tested: 50% (A,B), 60% (C,D), 70% (E,F) and 80% (G,H). A,C,E,G: percentages of duplicates in monogenic genes and non-disease genes. B,D,F,H: percentages of monogenic disease genes as function of number of duplicates in human; 0 indicates that genes are singletons (have no homologs in human).
(EPS)

Figure S2 The same as Figure 1, only the duplicated genes were detected using eggNOG3. A) percentages of duplicates in monogenic genes and non-disease genes. B) percentages of monogenic disease genes as function of number of duplicates in human; 0 indicates that genes are singletons.
(EPS)

Figure S3 A) Comparing with non-disease genes (NDs) of similar duplication age, monogenic disease genes (MDs) tend to have higher coexpression with their closest paralogs. B) the same is true when age is omitted. The expression data were obtained from [24].
(EPS)

Figure S4 The number of unique GO terms (Molecular Function) as a function of branch length in MD- and ND- pairs. A) number of GO terms associated with a pair of genes in each group as a function of duplication age. B) the same as A) but the age was omitted.
(EPS)

Figure S5 Higher functional similarities in MD pairs comparing with ND-pair using protein-protein interaction data. A) shared protein interaction partners of MD pairs were compared with ND-pairs of similar age. B) the same as A) but the age was omitted.
(EPS)

Figure S6 The same as Figure 4, only the outliers were removed from the plots. Compared with non-disease genes (NDs), disease genes tend to have lower dN values with their mouse- (A) and Macaca- (B) one-to-one orthologs. Furthermore, compared with disease singletons, duplicated disease genes tend to have lower dN values with their mouse- (C) and Macaca- (D) orthologs. The higher selective constraints on duplicated disease genes can be also seen within the human genome; for example, compared with duplicated non-disease genes (ND) of similar duplication age, disease genes tend to have lower dN values with their closest paralogs within human (E). However the same isn't true when age is omitted (F), highlighting the importance of dividing gene pairs according to their duplication age.
(EPS)

Figure S7 Dating duplication events by comparing the topologies of gene trees with a reference species tree. A) duplication event on a gene tree. B) the corresponding event mapped to a species tree.
(EPS)

Figure S8 The species tree used in this study. Highlighted in red is the time period during which the two rounds of whole genome duplications (WGDs) likely happened.
(EPS)

Dataset S1 This supplementary file is an archive contains all the raw data and R scripts used in this study; also included in this archive is a detailed instruction for the readers to reproduce our main results, including all the figures, supplementary figures, and statistical tests except Figure 5, which was plotted manually.
(ZIP)
